# Modeling and Control of an Active Stabilizing Assistant System for a Bicycle

**DOI:** 10.3390/s19020248

**Published:** 2019-01-10

**Authors:** Chih-Keng Chen, Trung-Dung Chu, Xiao-Dong Zhang

**Affiliations:** 1 Department of Vehicle Engineering, National Taipei University of Technology, Taipei 10608, Taiwan; t107669401@ntut.org.tw; 2 Department of Mechanical and Automation Engineering, Da-Yeh University, Changhwa 51505, Taiwan; tdungchu@gmail.com

**Keywords:** bicycle, gyroscopic effect, balancing assistance, model predictive control

## Abstract

This study designs and controls an active stabilizing assistant system (ASAS) for a bicycle. Using the gyroscopic effect of two spinning flywheels, the ASAS generates torques that assist the rider to stabilize the bicycle in various riding modes. Riding performance and the rider’s safety are improved. To simulate the system dynamic behavior, a model of a bicycle–rider system with the ASAS on the rear seat is developed. This model has 14 degrees of freedom and is derived using Lagrange equations. In order to evaluate the efficacy of the ASAS in interacting with the rider’s control actions, simulations of the bicycle–rider system with the ASAS are conducted. The results for the same rider for the bicycle with an ASAS and on a traditional bicycle are compared for various riding conditions. In three cases of simulation for different riding conditions, the bicycle with the proposed ASAS handles better, with fewer control actions being required than for a traditional bicycle.

## 1. Introduction

The modeling of bicycle rider control is a common research topic. Van Lunteren et al. [1] used classical control methods to model rider control in order to study the effect of drugs, alcohol, and other factors on the ability of the rider to stabilize a bicycle. In a control-theoretical study, Hess et al. [2] developed a bicycle rider model that is similar to an aircraft pilot model. This model includes gain values, a second-order filter, and a preview time.

Another approach to modeling rider control is optimal control. Schwab [3] used an LQR (Linear Quadratic Regulator) controller with full state feedback to determine the control that is required for a rider to stabilize a bicycle when leaning and steering. Sharp [4] also used LQR but for optimal control tracking with a road preview for a bicycle. Ryuma Hatano et al. [5] derived an accurate bicycle model that describes its characteristic behavior. A state-dependent Riccati equation (SDRE) control was applied to the model to achieve stabilization. David Rodriguez-Rosa et al. [1,6] proposed a design for an adaptive controller for an autonomous bicycle. The controller is robust to disturbances, such as wind or uneven surfaces, and minimizes the energy that is required for lateral stabilization of the bicycle. Amit Ailon et al. [7] proposed a control method that stabilizes the bicycle at a desired lean angle for a non-linear non-minimum phase model of a two-wheeled vehicle. Lyapunov stability theory is used to establish a continuous feedback loop for the steering input signal that obtains exponential stability at a desired lean angle.

In a review of the application of optimal control theory in vehicle dynamics, Sharp and Peng [8] noted that model predictive control (MPC) is a promising solution for modeling drivers and riders because its characteristics are pertinent to driving and riding strategies. This method accounts for the vehicle dynamics, the driver/rider skills that are associated with a system prediction model, and the variation in the cost function for the optimization problem. The view of the driver/rider while riding and the physical limitations of the situation are simulated by using preview reference information and the ability of MPC to deal with constraints. Dole et al. [9] used predictive control theory to model a car driver’s steering inputs during a yaw-sideslip incident. A comparison the results for an unconstrained MPC and a LQR with signal preview showed that MPC allows optimal control with an abbreviated prediction horizon.

For a single-track vehicle, Frezza et al. [10] used MPC to develop models of motorcycle racers. The models allow good control in simulating a set of more than 70 maneuvers with different vehicle models. Rowell et al. [11] made use of PC to model lane-change maneuvers for a motorcycle. The feasibility of predictive control was verified by the simulation results for various weighting matrices and preview distances.

Recently, Massaro [12,13] presented a virtual rider that used predictive control to guide a non-linear motorcycle along target profiles for roll angle and speed. The control inputs for this virtual rider are comparable to a real rider’s inputs, as measured during a real maneuver. Although MPC is suited to modeling both a car driver and a motorcycle rider, few studies have used MPC to model a bicycle rider.

There are many applications based on the gyroscopic effect in a large range of technical fields. This effect has been used for both active and passive stabilization of unstable dynamic systems due to its ability to generate a large torque and a fast response. Townsend and Shenoi [14] discussed the gyroscopic effect and applied it to stabilize a ship’s roll motion. In the field of aeronautical engineering, the gyroscopic effect is widely used to guide the altitudes of spacecraft and satellites in space [15,16]. In addition, Lemus et al. [17,18] conducted experiments using an inverted pendulum test bench instead of a real human to enhance human balance, which is also based on the gyroscopic effect.

Cycling contributes many benefits, such as being healthy and environment-friendly, as well as easing traffic congestion. Therefore, more governments are promoting and encouraging their citizens to cycle. However, cyclists have a relatively high injury risk for road crashes [19]. Hence, improving the bicycle’s stability is highly demanded and has become the subject of many studies. Steering using the handlebars [20,21] and the gyroscopic effect of spinning flywheels are used to stabilize a bicycle. P.Y. Lam [22] used a control moment gyro as an actuator to stabilize a small bicycle. By using a PD (Proportional and Differential) controller, the bicycle was successfully balanced and could move forward and backwards, and could turn through a small angle. H. Yetkin et al. [23] proposed an autonomous bicycle, and it was controlled by the gyroscopic effect of a flywheel.

A sliding mode control algorithm is applied for stabilization and has been experimentally proven to provide good performance. Hsieh [24] proposed a gyroscopic balancer for a rider-less bicycle that stabilizes a bicycle that is stationary or moving forward. In Reference [25], Chi extended this work by developing an adaptive fuzzy sliding mode controller for the balancer system. To develop an active safety system for single-track vehicles, Różewicz et al. [26] proposed a mathematical model of a bicycle that is steered by a control moment gyro.

A review of the research on the behavior of a bicycle under the gyroscopic effect of two spinning flywheels shows that an active stabilization assistant system used in single-track vehicles is possible. This system increases the bicycle’s stability and controllability and enhances the rider’s comfort and safety. If it is under the assumption that the rider’s body is fixed to the bicycle frame and that there is no slipping at the contact points between the road surface and the tires, the balancing problem for a bicycle is the same as the problem of balancing an inverted pendulum.

An inverted pendulum used as a benchmark for the balancing problem is common in studies that pertain to control theory, robotics, and vehicle engineering. In Reference [27], this method is used to simulate an electric bicycle with power steering systems. A gyroscopic inverted pendulum is applied to benchmark an adaptive control algorithm for a single-wheeled pendulum robot in Reference [28]. The experiments proved that the pendulum model with the single-wheel pendulum robot was valid. Similarly, an inverted pendulum is used to analyze the validity of a stabilizing device before applying it to a bicycle [29].

In a previous work by the authors, an active stabilizing assistant system (ASAS) for a bicycle that uses the gyroscopic effect of two flywheels was designed. A model predictive controller (MPC) was used to control the system and the efficacy of this controller was verified via experiment in References [21,30]. In order to determine the system’s performance, this study determines the efficacy of the ASAS when interacting with the rider’s control actions.

Using gyroscopic effects of spinning wheels to stabilize a bicycle was studied in various studies as described in the literature review. Since most of these researches aimed to develop a rider-less autonomous bicycle, the complex dynamic behaviors of the rider when controlling the bicycle are ignored. Although the bicycles in these literatures could be balanced at low speeds and under disturbances, there are difficulties in making a turn. In order to avoid a continuous rotation in the gimbal angles of the flywheels, the bicycles are kept upright and turned at a very low longitudinal speed. This affects the main advantage of single-track vehicles: high mobility. In this paper, we develop an ASAS to assist the riders in stabilizing the bicycles. In order to assert the assisting performances of the ASAS, interactions between the rider’s control actions and the bicycle are considered. The ASAS in this study is aimed to become integrated with a bicycle as a type of single-track vehicle with more stability and high mobility.

The rest of this paper is organized as follows. In Section 2, the system model of a bicycle with ASAS is presented. In Section 3, the control strategies for a bicycle–rider system with ASAS are described. Section 4 discusses the results of control simulations and Section 5 provides conclusions.

## 2. System Modeling

In this section, a dynamic model of a bicycle with a rider is introduced. The active assistant system modeling is then established using the gyroscopic effect. A complete model of the bicycle–rider system with ASAS is then developed to allow simulation.

### 2.1. Bicycle–Rider System

**Figure 1:**
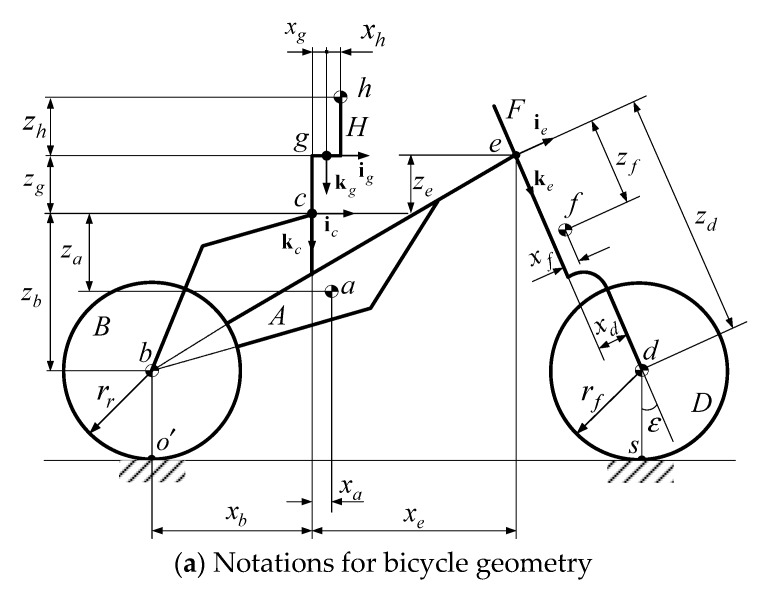

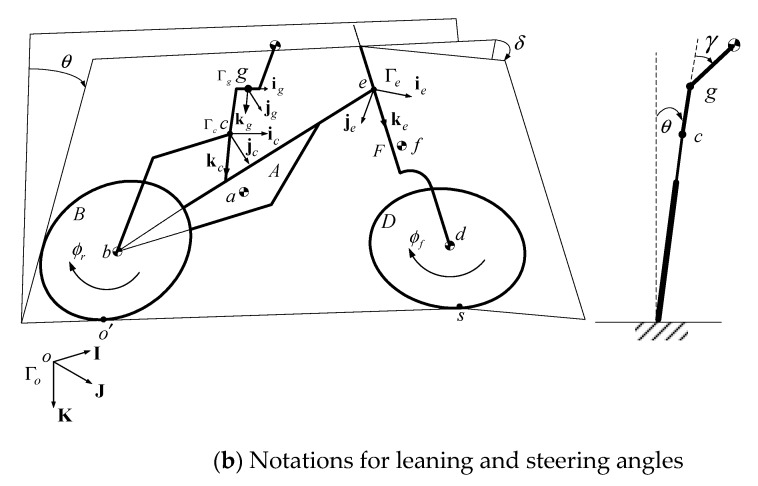
Scheme of the bicycle model.

Figure 1 shows the scheme of the bicycle–rider system. The four letters, A, B, D, and F, represent the four rigid bodies of the bicycle: the rear wheel, front wheel, the front forks, and the bicycle frame, respectively. The rider’s upper body, H, can lean on the hinge, g, relative to the bicycle frame. The center of mass of each rigid body is designated by the corresponding lower-case letters, a, b, d, f, and h.

Four coordinate systems (SAE, Society of Automotive Engineers, standard) are used in this model: an inertial frame that is fixed on the ground $\Gamma_{o}\left(I,J,K\right)$, a reference frame $\Gamma_{c}\left(i_{c},j_{c},k_{c}\right)$ at the saddle of the bike, a reference frame for the steering angle $\Gamma_{e}\left(i_{e},j_{e},k_{e}\right)$ at point e, and the hinge frame $\Gamma_{g}\left(i_{g},j_{g},k_{g}\right)$.

The relative positions and orientations of the bicycle to the inertial frame $\Gamma_{o}$ are described by the six coordinates of the reference point $\Gamma_{c}$: the position parameters (X,Y,Z) and the three 3-2-1 Euler angles (ψ,ϕ,θ). The steering angle δ is defined as the relative rotation of the frame $\Gamma_{c}\left(i_{c},j_{c},k_{c}\right)$ and $\Gamma_{e}\left(i_{e},j_{e},k_{e}\right)$. The angles of rotation of the front and rear wheels are represented by two parameters $\phi_{f}$ and $\phi_{r}$, respectively. The lean angle of the rider’s upper body is denoted as parameter γ. Using these definitions, the generalized coordinates $q\in {\mathbb{R}}^{10}$ are written as:

(1) $$q={\left[\begin{array}{cccccccccc} X & Y & Z & \psi & \phi & \theta & \delta & \phi_{r} & \phi_{f} & \gamma \end{array}\right]}^{T}.$$

The vector of velocity $u\in {\mathbb{R}}^{10}$

(2) $$u={\left[v_{x}\;v_{y}\;v_{z}\;\omega_{x}\;\omega_{y}\;\omega_{z}\;\dot{\delta }\;{\dot{\phi }}_{r}\;{\dot{\phi }}_{f}\;\dot{\gamma }\right]}^{T},$$

is defined as the quasi-velocities of the bicycle body in the reference frame $\Gamma_{c}$. u is related to the generalized velocities $\dot{q}$ by:

(3) $$u=Y\dot{q},$$

where

(4) $$Y=\left[\begin{array}{ccc} R_{c} & 0 & 0\\ 0 & S & 0\\ 0 & 0 & I \end{array}\right].$$

The transform matrix $Y\in {\mathbb{R}}^{10\times 10}$ is composed of the rotation matrix $R_{c}$ from $\Gamma_{o}$ to $\Gamma_{c}$, the transformation matrix for the Euler angles:

(5) $$S=\left[\begin{array}{ccc} -\sin\phi & 0 & 1\\ \cos\phi \sin\theta & \cos\theta & 0\\ \cos\phi \cos\theta & -\sin\theta & 0 \end{array}\right],$$

and the identity matrix $I\in {\mathbb{R}}^{4\times 4}$.

In the model of this two-wheeled vehicle, the contacts between the two wheels and the ground are assumed to have a rolling-without-slipping property. Using the relationships of kinematic contact between the wheels and the ground, four constraint equations can be derived for each wheel, where two are holonomic and two are non-holonomic.

**Figure 2:**
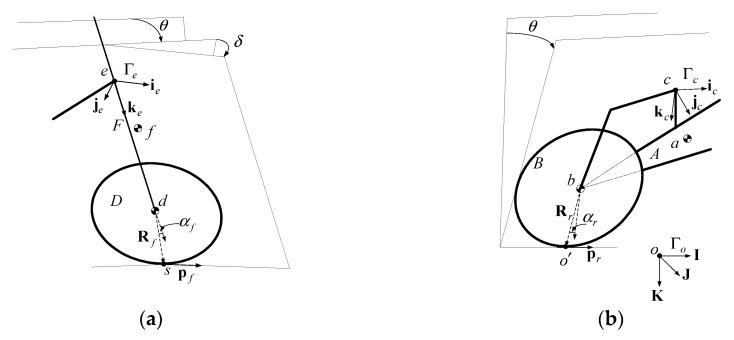
Scheme for (**a**) the rear wheel and (**b**) the front wheel.

Each contact point is calculated using a new algebraic variable: $\alpha_{f}$ for the front wheel and $\alpha_{r}$ for the rear wheel. Angle $\alpha_{f}$ is between $k_{e}$ and $R_{f}$ and $\alpha_{r}$ is between $k_{c}$ and $R_{r}$, as illustrated in Figure 2. The two algebraic variables extend the number of variables to 12, so the generalized-coordinate and quasi-velocity vectors are expanded to:

(6a) $$q_{e}={\left[\begin{array}{cccccccccccc} X & Y & Z & \psi & \phi & \theta & \delta & \phi_{r} & \phi_{f} & \gamma & \alpha_{r} & \alpha_{f} \end{array}\right]}^{T},$$

(6b) $$U={\left[\begin{array}{cccccccccccc} v_{x} & v_{y} & v_{z} & \omega_{x} & \omega_{y} & \omega_{z} & \dot{\delta } & {\dot{\phi }}_{r} & {\dot{\phi }}_{f} & \dot{\gamma } & {\dot{\alpha }}_{r} & {\dot{\alpha }}_{f} \end{array}\right]}^{T}.$$

All the holonomic constraints are differentiated to yield the velocity form, which, together with other non-holonomic constraints, can be written in matrix form as:

(7) $$B_{e}U=0$$

where $B_{e}\in {\mathbb{R}}^{8\times 12}$ is the constraint Jacobian matrix. The equations of motion with constraint conditions then become:

(8) $$\left\{ \begin{array}{l} {\dot{q}}_{e}=W_{e}U,\\ J_{e}\dot{U}=Q_{e}-B_{e}^{T}\lambda \\ B_{e}U=0. \end{array}\right.,$$

where $W_{e}\in {\mathbb{R}}^{12\times 12}$ is defined as $\left[\begin{array}{ccc} W & 0 & 0\\ 0 & 1 & 0\\ 0 & 0 & 1 \end{array}\right]$, $J_{e}\in {\mathbb{R}}^{12\times 12}$ as $\left[\begin{array}{ccc} J & 0 & 0\\ 0 & 0 & 0\\ 0 & 0 & 0 \end{array}\right]$, $Q_{e}\in {\mathbb{R}}^{12}$ as $\left[\begin{array}{c} Q\\ 0\\ 0 \end{array}\right]$, and $\lambda \in {\mathbb{R}}^{8}$ represents the eight Lagrange multipliers or constraint forces that are coupled to the system by the constraint Jacobian matrix $B_{e}$. The details of the derivation of the equations of motion for the bicycle–rider system are given in Reference [21]. The parameters for the bicycle–rider system that are used in the simulations are shown in Table 1 and Table 2.

### 2.2. Modeling the Active Stabilizing Assistant System

**Figure 3:**
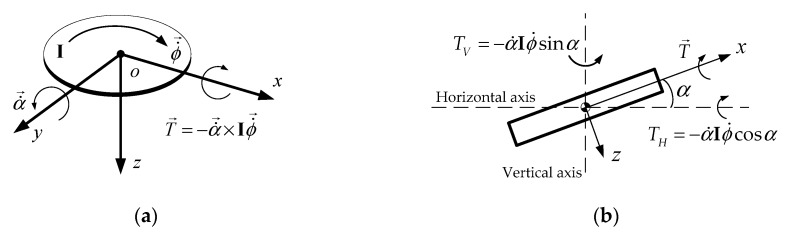
The principle for the design: (**a**) the gyroscopic effect of a spinning disk and (**b**) the effective torques around the horizontal and vertical axes due to the gyroscopic effect [30].

In previous work [30], a gyroscopic system used according to the gyroscopic effect of a spinning disk is developed, as shown in Figure 3. A disk with moment of inertia I spins about its z-axis at a speed of $\vec{\dot{\phi }}$. When its precession angular rate $\vec{\dot{\alpha }}$ is controlled, a torque is generated along its x-axis with its magnitude and direction defined as:

(9) $$\vec{T}=-\vec{\dot{\alpha }}\times I\vec{\dot{\phi }}.$$

At a precession angle α, the generated torques around the horizontal and vertical axes are:

(10) $$T_{H}=-\dot{\alpha }I\dot{\phi }\cos\alpha ,$$

(11) $$T_{V}=-\dot{\alpha }I\dot{\phi }\sin\alpha .$$

where $T_{H}$ is used to stabilize the vehicle system in the direction of roll while $T_{V}$ causes uncontrolled yaw motion for the vehicle which is not required.

If two flywheels rotate in opposite directions at the same speed, the torque on the vertical axis of a flywheel cancels with each other. Since the magnitude of $T_{H}$ depends on the cosine of precession angle α, it should be as small as possible to generate a larger effective torque along the horizontal axis. At a precession angle of ±90°, the effective torque on the horizontal axis is zero.

**Figure 4:**
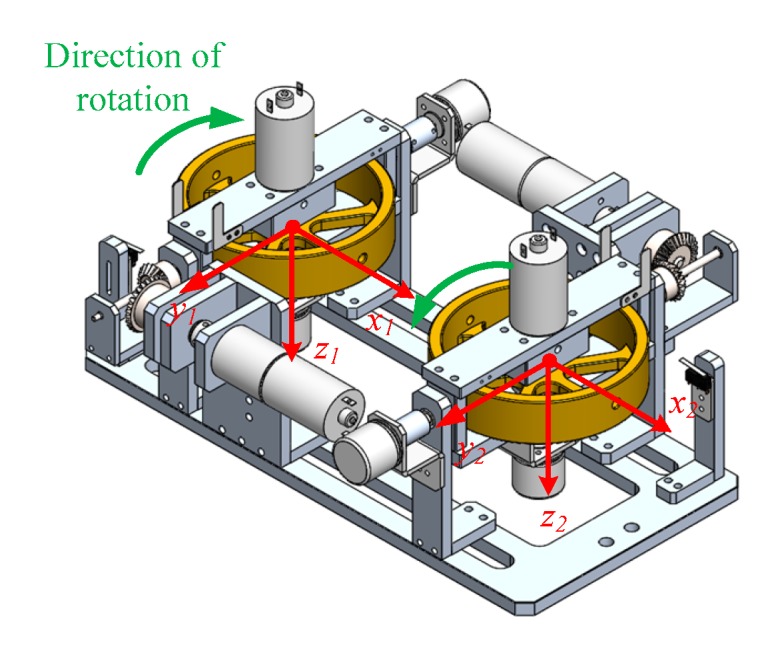
The directions of rotation for the flywheels.

Figure 4 shows the proposed ASAS with two spinning flywheels attached to a gimbal frame including four electric motors and bevel gears. The two flywheels spin in opposite directions to cancel the unrequired effect of a rotating mass on the yaw dynamics of the vehicle. If there are two flywheels, the maximum effective torque generated is the summation of the torque from each flywheel.

### 2.3. Bicycle–Rider System with ASAS

**Figure 5:**
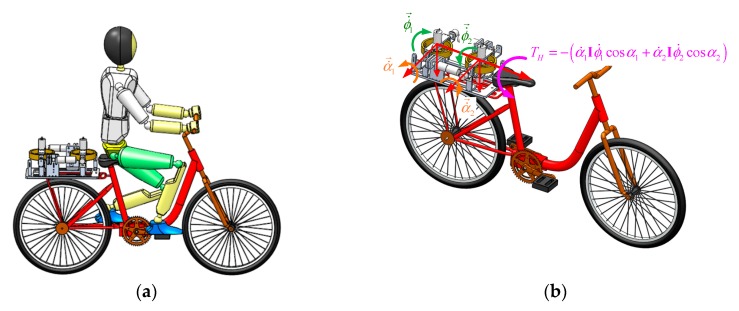
Bicycle–rider system with an ASAS and the assistant torque that acts on the bicycle: (**a**) bicycle–rider with ASAS and (**b**) the assistant torque that acts on the bicycle.

A model of a bicycle-rider system with an ASAS on the rear seat is shown in Figure 5a. By rotating the gimbal angles of the two flywheels, the ASAS generates an assistant torque that allows the rider to stabilize the bicycle. The magnitude of the assistant torque in the horizontal direction of the bicycle is the sum of the torques that are generated by each flywheel

(12) $$T_{H}=-\left({\dot{\alpha }}_{1}I{\dot{\phi }}_{1}\cos\alpha_{1}+{\dot{\alpha }}_{2}I{\dot{\phi }}_{2}\cos\alpha_{2}\right).$$

**Figure 6:**
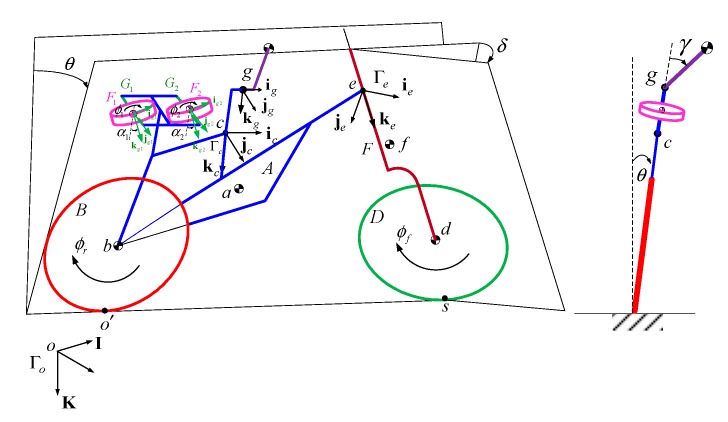
A scheme for a rider with an assisted bicycle.

The dynamic model of the bicycle-rider with an ASAS is derived using the equations of motion from Section 2. The ASAS with four bodies—D1, D2, F1, F2—is attached to the rear seat, as shown in Figure 6. The original number of degrees of freedom for the bicycle is increased from ten to fourteen. The coordinates of the bicycle still use the reference point c, between the seat and the vehicle body. Three new SAE (Society of Automotive Engineers) standard coordinate systems are used in the model: a frame $\Gamma_{g}\left(i_{g},j_{g},k_{g}\right)$ is used to describe the motion of the rider’s upper body, and $\Gamma_{g1}\left(i_{g1},j_{g1},k_{g1}\right)$ and $\Gamma_{g2}\left(i_{g2},j_{g2},k_{g2}\right)$ are used to describe the gimbal angles and the rotational motions of the stabilizing system’s flywheels.

Using the dynamic model of the bicycle–rider system, the motion of the new model is described in terms of the motion of reference point c as shown in Figure 1. Four additional coordinate variables, $\phi_{1},\;\phi_{2},\;\psi_{1},\;\psi_{2}$, which are related to the gimbal angles and the angles of rotation of the ASAS, are added to the generalized coordinates q. The equation of motion is then written as:

(13) $$q={\left[\begin{array}{cccccccccccccc} X & Y & Z & \psi & \phi & \theta & \delta & \phi_{r} & \phi_{f} & \gamma & \alpha_{1} & \phi_{1} & \alpha_{2} & \phi_{2} \end{array}\right]}^{T}.$$

The quasi-velocity vector is also expanded to $u\in {\mathbb{R}}^{14}$:

(14) $$u={\left[\begin{array}{cccccccccccccc} v_{x} & v_{y} & v_{z} & \omega_{x} & \omega_{y} & \omega_{z} & \dot{\delta } & {\dot{\phi }}_{r} & {\dot{\phi }}_{f} & \dot{\gamma } & {\dot{\alpha }}_{1} & {\dot{\phi }}_{1} & {\dot{\alpha }}_{2} & {\dot{\phi }}_{2} \end{array}\right]}^{T}.$$

The Lagrange equation is used to derive the system’s equations of motion:

(15) $$\frac{d}{dt}\left(\frac{\partial T}{\partial u}\right)+\frac{\partial T}{\partial u}\Delta -\frac{\partial T}{\partial q}W+\frac{\partial V}{\partial q}W=U_{nc}^{T},$$

where the total kinetic energy is $T=\frac{1}{2}u^{T}\mathrm{Ju}$ and the inertial matrix of the system is $J\in {\mathbb{R}}^{14\times 14}$. The total potential energy, $V=\sum_{i}^{9}-m_{i}gZ$ and $Z_{i}$ are the vertical coordinates of the corresponding bodies in the inertial frame $\Gamma_{o}$. The coefficient matrices Δ and W are expanded to ${\mathbb{R}}^{14\times 14}$ when the identity matrix is replaced by $I\in {\mathbb{R}}^{8\times 8}$.

**Figure 7:**
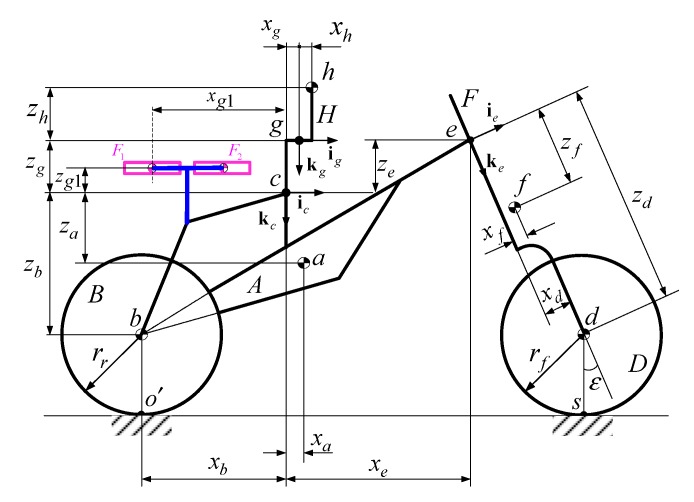
The system’s dimensions.

The parameters for the system in Figure 7 are shown in Table 3. These parameters are obtained by dimensioning the center of mass of the bicycle bodies using the ASAS system on the rear seat.

## 3. Control Strategies

The MPC1 controller that uses a rider model is constructed and the MPC2, which is used to control the active assistant system, is described. The control of the bicycle–rider system with an ASAS with two MPCs is then described.

### 3.1. Rider Control Model

**Figure 8:**
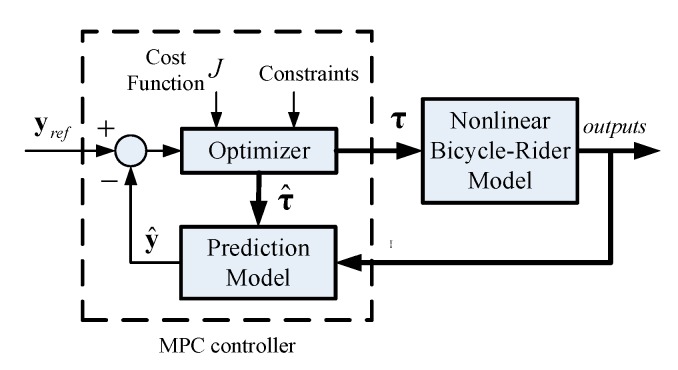
Control diagram for the MPC1 controller.

When riding a bicycle, a rider can predict how control actions will affect the bicycle’s state, based on practical experience of the system’s physical characteristics and dynamics. MPC with a control output is optimized using the prediction of the system’s future behavior and constraints. This allows modeling of the system whereby a rider controls a bicycle. The block diagram for an MPC is shown in Figure 8. Using a prediction model, the MPC calculates the system’s future outputs $\hat{y}$ to a set of future inputs $\hat{\tau }$. The future inputs are generated via an optimization process, which minimizes the quadratic cost function J for the predicted errors and the control efforts. The physical limitations of the system are modeled as constraints in the optimization process. The rider model for this study uses an MPC to track the roll angle. The torque that acts on the bicycle’s front forks dues to steering inputs at the handlebars and the leaning torque of the rider’s upper body are the control inputs.

The continuous time linear model in state-space form is:

(16) $$\begin{array}{l} \dot{x}=\mathrm{Ax}+B\tau ,\\ y=\mathrm{Cx}, \end{array}$$

where **x** is the state vector; ***y*** is the output; **τ** is the control input; and **A**, **B**, and **C** are the coefficient matrices.

A discrete linear model of the bicycle–rider system is used to predict the system’s response:

(17) $$\begin{array}{c} x(k+1)=A_{d}x(k)+B_{d}\tau (k),\\ y(k)=C_{d}x(k), \end{array}$$

where the discrete state-space matrices—$A_{d}$, $B_{d}$, and $C_{d}$—are easily computed using the continuous linear model (16) with discrete sampling $T_{s}$.

The control torque sequence is the result of an optimization problem, which minimizes the quadratic cost function:

(18) $$J\left(k\right)=\sum_{i=1}^{m}{\left\|y_{ref}\left(k+i\right)-y\left(k+i\right)\right\|}_{Q}^{2}+\sum_{i=0}^{n-1}{\left\|\tau \left(k+i\right)\right\|}_{R}^{2},$$

with the constraints

(19) $$\tau_{\min}\leq \tau \left(k+i\right)\leq \tau_{\max},$$

where $y_{ref}$ is the reference signal and $\tau_{\min}$, and $\tau_{\max}$ are the maximum and minimum control torques that the rider generates.

### 3.2. ASAS Control

The ASAS uses two flywheels that spin at a constant speed in opposite directions. When the gimbal angles change at a rate of ${\dot{\alpha }}_{1}$ and ${\dot{\alpha }}_{2}$, the gyroscopic effect generates torque at the pivot of the inverted pendulum, which stabilizes the system, as shown in Equation (9). To make a summation of the effective torque, the gimbal angles $\alpha_{1}$ and $\alpha_{2}$ must be in opposite directions.

The main tasks of the MPC are to control the rotation of the gimbal frames that generate the torque that allows the angle θ to follow the reference signals. However, since Equation (12) uses the cosine of the gimbal angles, MPC must ensure that $\alpha_{1}$ and $\alpha_{2}$ are as small as possible to ensure the largest control torque. When θ reaches the value of the reference, MPC must return the gimbal angle to its zero position, to prepare for the next action. Therefore, the control problem for this system has two control inputs and three control outputs.

Using the prediction model, MPC computes the optimal control sequences for the gimbal torque to minimize the first term of the cost function (Equation (18)). The accuracy of control and the robustness of MPC are affected by the parameters in Equation (18). Using larger weights for the weighting matrix Q increases the controller’s ability to track errors (the tracking errors are decreased), but more gimbal torque is required from the electric motors. Increasing the value of the weighting matrix R gives a higher priority to reducing the control efforts and results in less accurate tracking. The other parameters for MPC are the prediction horizon *m* and the control horizon *n*. A smaller value *m* of gives a more aggressive controller. However, as noted by Wojsznis et al. [31], a large prediction horizon means that a further increase has a minor effect on the control accuracy.

MPC uses a model of the plant to predict future behavior. The model is discrete and linear time-invariant (LTI), and the state-space form is given by:

(20) $$\begin{array}{c} x(k+1)=\mathrm{Ax}(k)+\mathrm{Bu}(k)\\ y(k)=\mathrm{Cx}(k)+\mathrm{Du}(k) \end{array}$$

If an external force acts on the system and its magnitude is measurable, the effect of this force on the system’s dynamics is predicted by adding a term of measured disturbance into the prediction model. The linear prediction model (20) then becomes:

(21) $$\begin{array}{c} x(k+1)=A_{d}x(k)+B_{d}\tau (k)+B_{v}v(k),\\ y(k)=C_{d}x(k), \end{array}$$

where $B_{v}$ is the disturbance parameter matrix and v(k) describes the measured disturbance.

**Figure 9:**
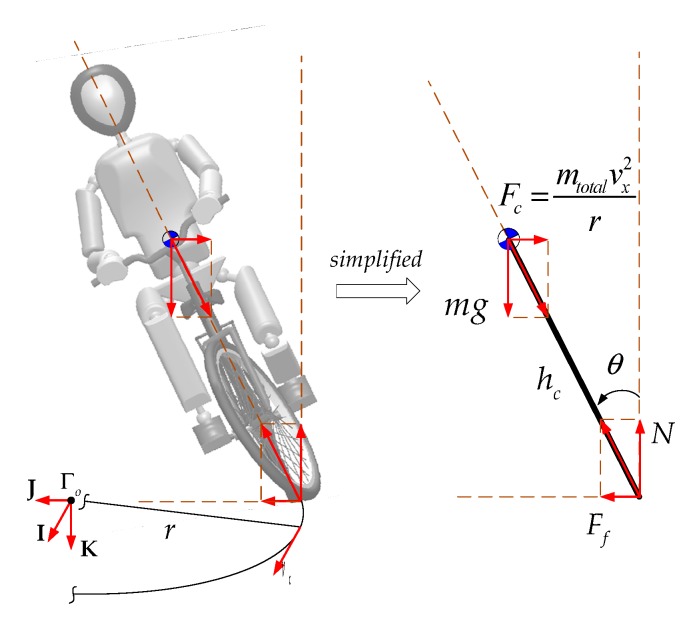
The forces that act on a bicycle during cornering.

**Figure 10:**
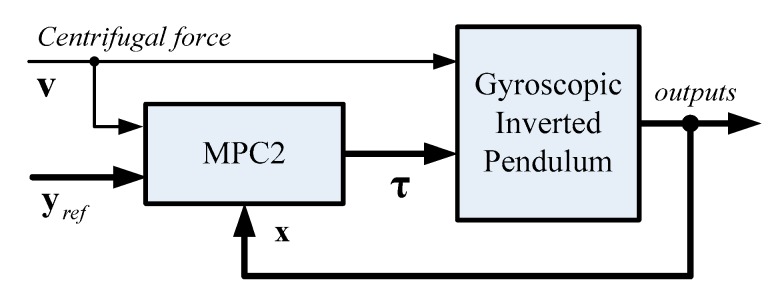
A block diagram for MPC2 with a measured disturbance.

Figure 9 demonstrates the forces that act on a bicycle–rider system when the bicycle goes into a constant-radius corner. The centrifugal force has an effect on the system’s dynamics, so the predicted values for future outputs take this into account by using the prediction model in Equation (21). The control scheme for this case is shown in Figure 10. The centrifugal force acts on the system’s center of mass and is given in the form of a measured disturbance v(k).

### 3.3. Control of a Bicycle–Rider System with ASAS

**Figure 11:**
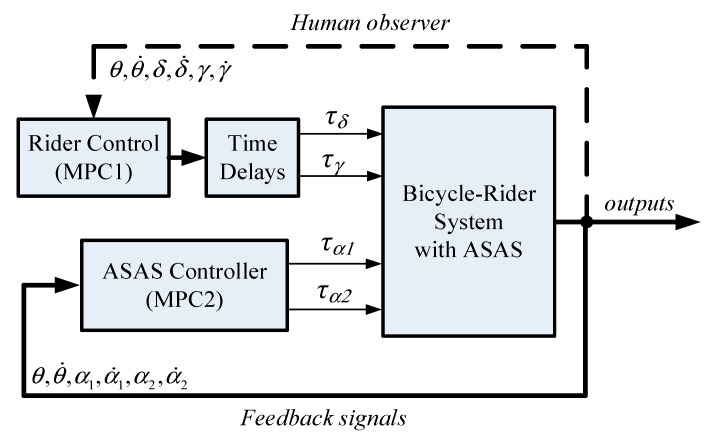
Control block diagram for an assisted bicycle with a rider.

This study uses two mode predictive controllers (Figure 11). MPC1 is used to model the rider input to control the bicycle using steering and leaning torques. MPC2 enables the ASAS system to generate an assistant torque that allows the rider to stabilize the bicycle.

To synthesize two MPCs, linear prediction models must be devised. MPC1 uses the system identification method that is described in Section 2. A non-linear model of the bicycle–rider system with an ASAS is used to generate the identification data. The Linear model for the first MPC is described in sixth order state-space form as:

(22) $$\begin{array}{c} A_{1}=\left[\begin{array}{cccccc} 0 & 1 & 0 & 0 & 0 & 0\\ 8.81 & -0.56 & -18.01 & -3.74 & 18.92 & 1.70\\ 0 & 0 & 0 & 1 & 0 & 0\\ 116.49 & 7.24 & -136.59 & -41.18 & 41.81 & 3.08\\ 0 & 0 & 0 & 0 & 0 & 1\\ -5.67 & 0.41 & 11.59 & 1.28 & -90.21 & -9.00 \end{array}\right],\\ B_{1}=\left[\begin{array}{cc} 0 & 0\\ -17.89 & -3.53\\ 0 & 0\\ 89.99 & -16.04\\ 0 & 0\\ 8.93 & 14.69 \end{array}\right]. \end{array}$$

**Figure 12:**
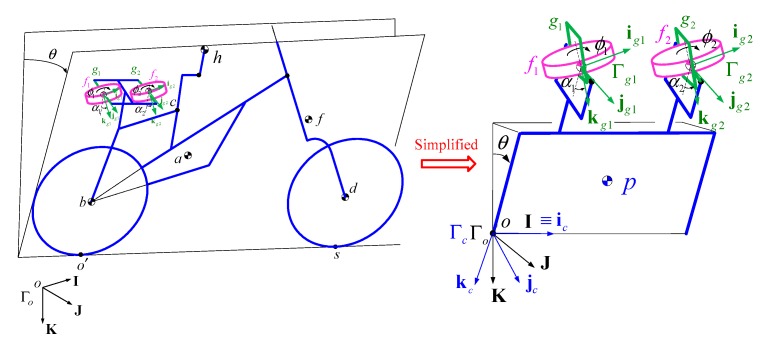
Simplified model of a bicycle–rider system with an ASAS.

The second MPC uses a nonlinear model that describes the bicycle–rider system with an ASAS as an inverted pendulum, whereby the rider’s body is rigidly attached to the bicycle frame and the steering for the bicycle is fixed, as shown in Figure 12. The linear equation of motion and the operating point for the system is detailed in Reference [30]. The numerical results for the linear prediction model for the second MPC are:

(23) $$\begin{array}{c} A_{2}=\left[\begin{array}{cccccc} 0 & 12.05 & -0.06 & 0 & 0.06 & 0\\ 1 & 0 & 0 & 0 & 0 & 0\\ 331.09 & 0 & 0 & -2.35 & 0 & 0\\ 0 & 0 & 1 & 0 & 0 & 0\\ -331.09 & 0 & 0 & 0 & 0 & -2.35\\ 0 & 0 & 0 & 0 & 1 & 0 \end{array}\right];\\ B_{2}=\left[\begin{array}{cc} 0 & 0\\ 0 & 0\\ 84.99 & 0\\ 0 & 0\\ 0 & 84.99\\ 0 & 0 \end{array}\right]. \end{array}$$

In reality, the rider’s control actions are limited by the physical limitations of the human body. In order to model the rider input that is necessary in terms of the limitations of the control frequency, the magnitude of the control torque, and the time delays for the control output due to the lag in human’s muscles, Massaro used experiments with real riding maneuvers [13] and found that a rider’s control are mostly made at a frequency of 2 Hz. Other experiments using bicycle riders showed that the steering torque does not exceed 5 Nm [32] and the leaning torque is 30 Nm [33] during normal riding. Kiewiet [33] studied the upper limb muscles of 15 young adults and found that there is a time delay for the steering action of about 0.2 s. However, the time delay for leaning is slightly less than that for a steering action [1]. To model the rider control at 2 Hz, the MPC1 uses a sampling time of 0.5 s and the hard constraints on the control output are written as:

(24) $$\begin{array}{l} 5\;\mathrm{Nm}\leq \tau_{\delta }\leq 5\;\mathrm{Nm},\\ 30\;\mathrm{Nm}\leq \tau_{\gamma }\leq 30\;\mathrm{Nm}, \end{array}$$

The lag in the rider’s muscles is modeled by using time delays in the control output of MPC1. The delay time for steering control is 0.2 s and for leaning control is 0.15 s.

## 4. Results and Discussions

### 4.1. Stabilizing and Disturbance Rejection

In the simulations, the rider control model (MPC1) used both steering and leaning torque to stabilize the bicycle from an initial angle. Similar to simulation 1, the results for a bicycle with an ASAS and a traditional bicycle that have the same rider are compared. The parameters for MPC1 for the rider control model are:

(25) $$Q_{1}=\left[\begin{array}{ccc} 1000 & 0 & 0\\ 0 & 0 & 0\\ 0 & 0 & 0 \end{array}\right],\;R_{1}=\left[\begin{array}{cc} 1 & 0\\ 0 & 1 \end{array}\right],$$

with a prediction horizon m=60 and a control horizon n=2.

**Figure 13:**
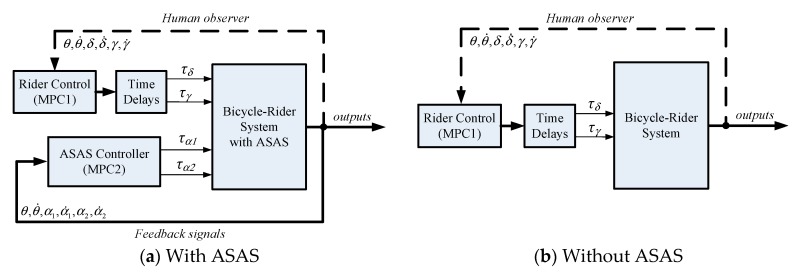
Block diagram for simulation.

**Figure 14:**
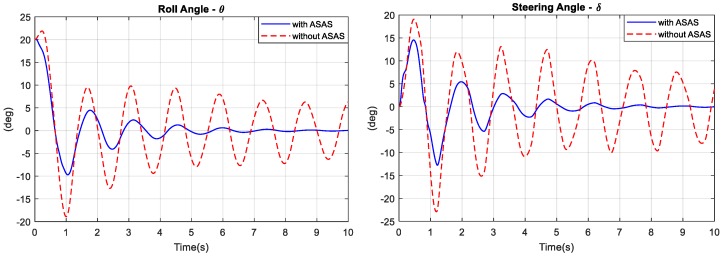

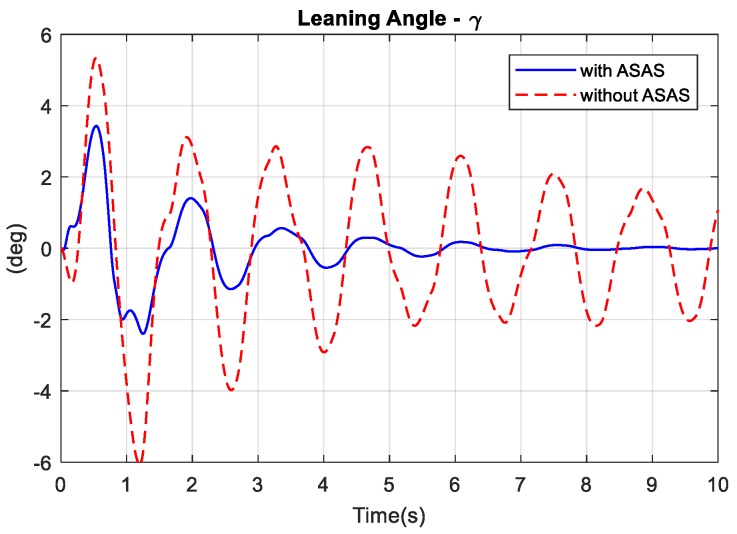
Balancing simulation: Angular responses.

**Figure 15:**
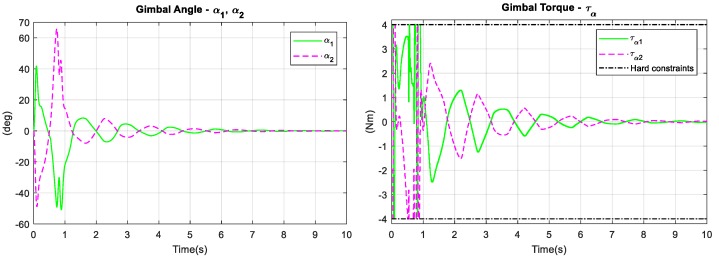
Balancing simulation: Gimbal angles and control torque.

**Figure 16:**
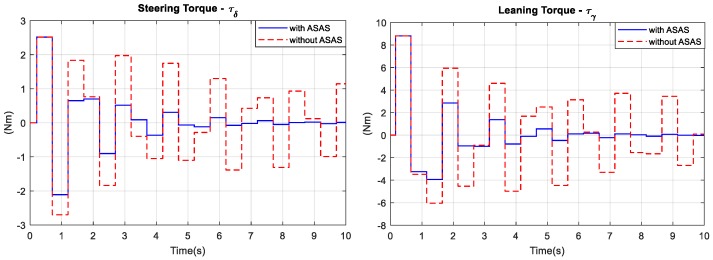
Control torque.

The bicycles have initial conditions of $v_{x}=15\;\mathrm{Km}/h$, $\theta \left(0\right)=20^{\circ }$. The block diagrams for both bicycle models are shown in Figure 13. Figure 14 compares the angular responses for both bicycle models. Figure 15 shows that to balance from an initial roll angle of 20°, the rider of the bicycle with an ASAS required 10 s, and the rider with a traditional bicycle oscillated at an amplitude of 5°. The control effort (two control torques) that was required to balance the bicycle with an ASAS was significantly reduced after 1.5 s as shown in Figure 16. Figure 15 shows the gimbal angles and the control torques for the ASAS. The maximum gimbal angles for this simulation do not exceed over 63°. In the first 1.5 s of the transient time, the ASAS required more control torque to rotate the gimbal at high speed and then generated a larger assistant torque. If the maximum torque of the electric motor was considered as a hard constraint in the online optimization problem, MPC2 performed well when the control torque was saturated at the limit. After 8 s, the gimbal angles were at the angle of the zero position and the control torque was zero.

**Figure 17:**
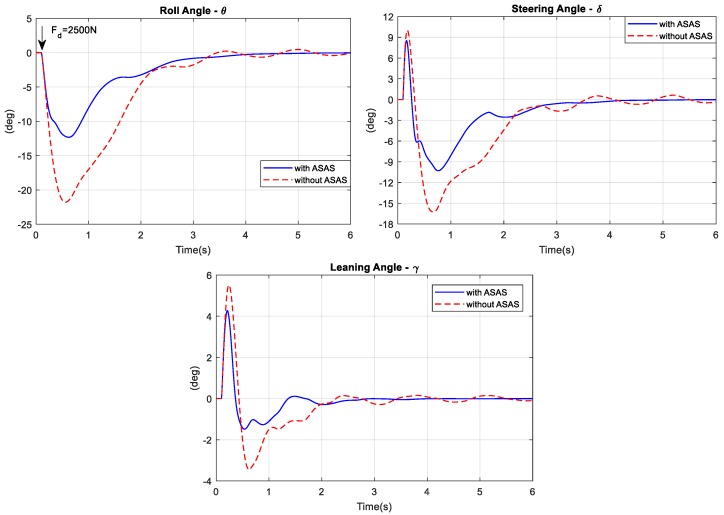
Disturbance rejection simulation: Angular responses.

**Figure 18:**
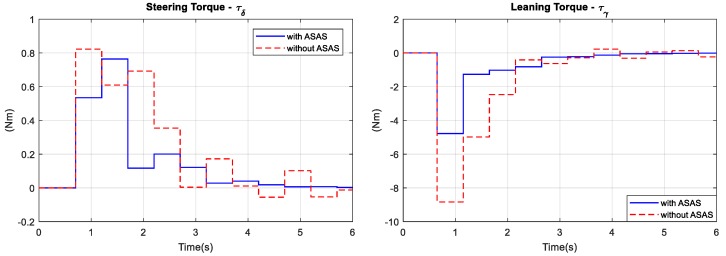
Disturbance rejection simulation: control torque.

To determine the bicycle’s ability to reject disturbance, the rider was simulated as riding the bicycle in a straight line at a speed of 15 km/h. As shown in Figure 17, at 0.1 s, an impulse disturbance acted on the seat and pushed the bicycle laterally. The amplitude of the disturbance was Fd = 2500 N. The disturbance pushed the bicycle to the left at a large roll angle. After 6 s, the rider stabilized both bicycle models. However, the maximum respective values of with and without the ASAS were 12° and 21°. Similar to the previous simulation, Figure 18 shows the rider’s control efforts were smaller when there was an ASAS.

### 4.2. Roll-Angle Tracking Control

**Figure 19:**
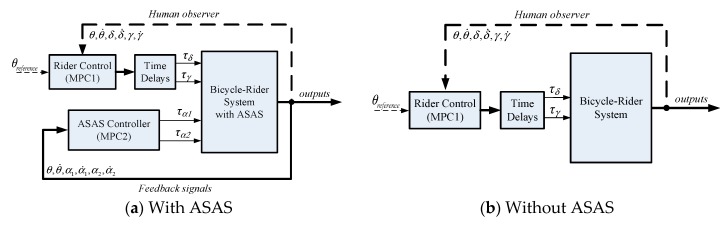
Control diagram for the simulation to track the roll angle.

Simulation 3 verifies the performance of the bicycle when the rider tracked a roll angle to change lanes or turn. Figure 19 shows that using steering and leaning torques, the rider ensured that the bicycle followed the reference signal for roll angle $\theta_{reference}$. In this case, the rider controlled the bicycle such that it achieved a roll angle that allowed it to follow the reference signal that is detailed in Reference [30] with an amplitude of 15°.

**Figure 20:**
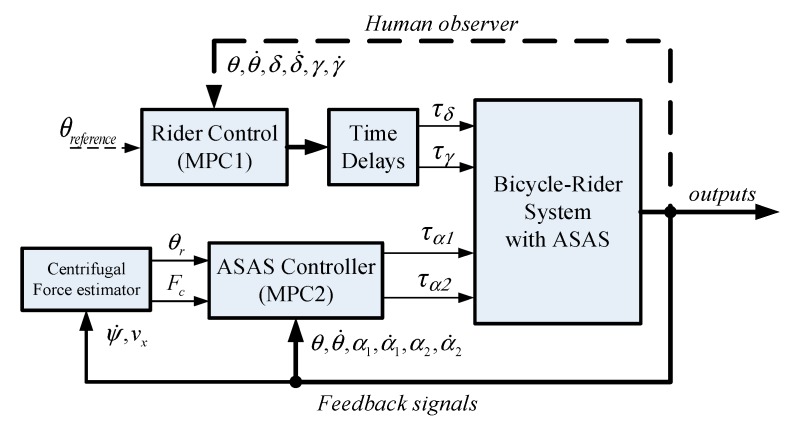
Control diagram for the ASAS to compensate for centrifugal force.

During cornering, a centrifugal force acts on the bicycle to change the trajectory (as shown in Figure 9). Stable cornering requires a roll angle that allows the bicycle to use its total weight to counteract the centrifugal force that arises during turning. The control diagram for the ASAS in Figure 19a did not account for the effect of the centrifugal force on the bicycle’s dynamics. An ASAS controller that compensated for the centrifugal force that acts during turning is shown in Figure 20 using the yaw rate and the longitudinal speed $v_{x}=\dot{\psi }r$ of the bicycle, the centrifugal force is calculated as:

(26) $$F_{c}=\frac{m_{total}{v_{x}}^{2}}{r}=\frac{m_{total}v_{x}\dot{\psi }r}{r}=m_{total}v_{x}\dot{\psi },$$

Using the same approach as Reference [30], the reference angle for MPC2 is estimated as:

(27) $$\theta_{r}=\mathrm{atan}\frac{v_{x}^{2}}{rg}=\mathrm{atan}\frac{v_{x}\dot{\psi }r}{rg}=\mathrm{atan}\frac{v_{x}\dot{\psi }}{g}.$$

The calculated centrifugal force is fed into MPC2 as a measured disturbance (MD), as shown in Figure 9, to allow a more accurate prediction of the behavior of the bicycle during a turn.

**Figure 21:**
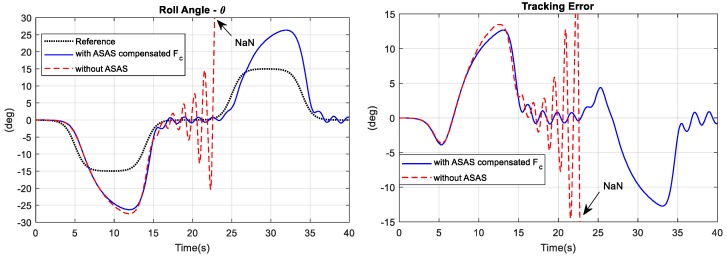
Comparison of the responses for a bicycle with an ASAS that compensates for the centrifugal force and those for a traditional bicycle.

To determine the performance of the ASAS with compensation for the centrifugal force, the simulation results for this controller were compared with those for a traditional bicycle under the same simulation conditions. The continuous line in Figure 21 shows the response for a bicycle with an ASAS and compensation for centrifugal force and the dashed line shows the response for a traditional bicycle. During the first 15 s of the simulation, the responses for both models were very similar, in terms of the roll angle, the steering angle, and the lean angle. The roll angle tracking error for the bicycle with an ASAS and compensation for the centrifugal force was slightly less than that for a traditional bicycle at a time of 12 s. However, after 15 s of tracking, the traditional bicycle begins to become unstable, and at 22 s, the bicycle was out of control and falls. The numerical value of the roll angle was not a finite number (NaN—not a number). The bicycle with compensation for the centrifugal force still tracked the reference signal with the same accuracy that it demonstrated in the first 15 s. The traditional bicycle went out of control because there were large time delays in the rider’s control output such that the rider could not follow the changes in the reference signal. The ASAS with compensation for the centrifugal force slowed the dynamics of the bicycle such that it was easier for the rider to control it.

**Figure 22:**
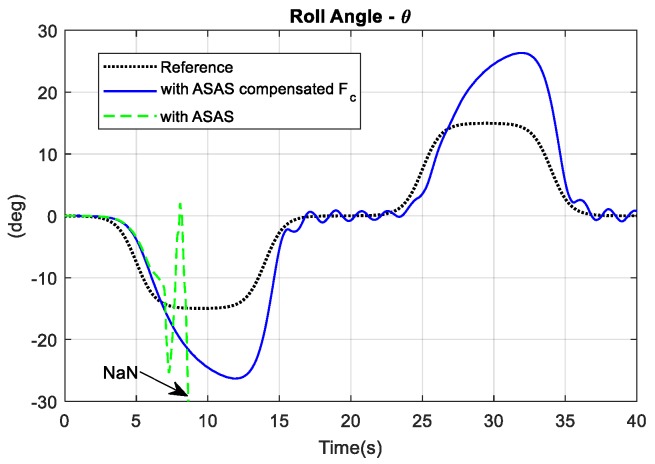
Comparison of the response for an ASAS that compensates for the centrifugal and that for a conventional ASAS.

Another simulation under the same conditions used a bicycle with an ASAS that compensated for centrifugal force and a bicycle with a conventional ASAS. In Figure 22, the continuous lines show the response for an ASAS that compensated for the centrifugal force and the dashed-dot lines show the response for a conventional ASAS. The simulation results showed that the normal ASAS exhibited poor dynamic behavior and was more difficult to control than a traditional bicycle. Eight seconds into the simulation, the bicycle with ASAS went out of control. The values of the roll angle, the steering angle, and the lean angle were not a finite number (NaN). If there was no compensation for centrifugal force, MPC2 predicted incorrect behavior for the bicycle such that the gimbal angles rotated continuously in one direction. The gyroscopic torque in this case acted as a disturbance to the rider’s control efforts.

While other methods [22,23,24,25] showed good performances toward stabilizing the bicycle at its upright positions, the turning motions seemed unnatural since the roll angle of the bicycle was kept as zero all the time to avoid the continuous rotating of the flywheel’s gimbal angle. Also, the steering was performed when the bicycle was at very low speed. Results of this research showed an important contribution of the proposed ASAS with a model predictive controller. In this case, the interactions between the ASAS and rider’s actions when controlling the bicycle to make a turn were studied. By using the centrifugal force that appeared during the corning as an information to predict the system behaviors, MPC successfully assisted the rider in making a required roll angle for turning.

### 4.3. Low Speed Stabilization

Controlling a bicycle at a low speed is very difficult because the system is highly unstable. Simulation 4 compares the stability and controllability of the two bicycle models at low speed. In this simulation, the rider must balance the bicycle at a longitudinal speed of 5 km/h. The block diagram in Figure 13 was used again. After 1 s, a 1000 N lateral disturbance acted at point c and the simulation proceeded for 5 s.

**Figure 23:**
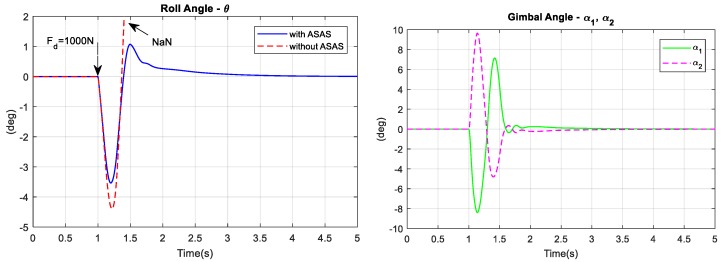
The simulation result for low speed stabilization.

Figure 23 shows the results for Simulation 4. The plot of roll angle versus time shows that even at a low longitudinal speed, the rider of the assisted bicycle could balance the bicycle after 5 s. When a disturbance acted on the system, the rider of the conventional bicycle gradually lost control of the bicycle and fell after a few seconds.

The maximum values for the gimbal angles during the simulation were about 10° and the maximum values for the gimbal torque was 1.8 Nm. After 3 s, the gimbal angles were at the zero position.

## 5. Conclusions

This study proposed a design for an active stabilizing assistant system (ASAS) for a bicycle. Using the gyroscopic effect of two spinning flywheels, the ASAS generated assistant torque that allowed the rider to stabilize the bicycle in various riding modes. The dynamic behavior of a bicycle–rider system with an ASAS on the rear seat was simulated using a mathematical model. This model has 14 degrees of freedom and was derived using Lagrange equations, with holonomic and non-holonomic constraints on the bicycle’s wheels.

Model predictive controllers (MPCs) were used to control the ASAS. This constituted a three-input, three-output control problem with hard constraints because of the physical limitations of the system. Using an inverted pendulum model for a bicycle-rider system, control strategies for different riding situations were proposed.

The efficiency of the ASAS in terms of its interaction with the rider’s control actions were determined using four simulations of the bicycle–rider system with an ASAS. For each simulation, a rider control model was developed using an MPC with time delays for the control output. The simulation results for the same rider model and various riding conditions for a bicycle with an ASAS and a traditional bicycle were compared. In six simulations using different riding conditions, the bicycle with the proposed ASAS had better performances and required fewer control actions than a traditional bicycle with the same rider.

The results of this research show the potential of the ASAS in developing a new type of single-track vehicles. By actively controlling two counter-rotating flywheels with a model predictive controller, the ASAS could improve the stability of the vehicles while not affecting the single-track vehicles’ mobility regarding turning and vehicle sizes. In situations that may cause losing control and taking a fall of the riders, such as when impacted by an external collision force or at very low speeds, the ASAS can assist the rider to stabilize the vehicle as an active safety system.

To ride a bicycle with an ASAS, a new bicycle rider must be trained, which is similar to the case for a traditional bicycle. Since the dynamic behavior of a bicycle is changed when an ASAS is installed, even an experienced rider requires time to become familiar with it.

In order to make the bicycle with the ASAS easier for new riders to use and to decrease the time that is taken to become familiar with the system, an adaptive model predictive control algorithm will be used in the future. Using adaptive controllers, an ASAS can adapt and generate an assistant torque more efficiently for riders with different physical characteristics and skill levels and for different types of bicycle.

## Figures and Tables

**Table 1:** Simulation parameters of bicycle–rider model.

Parameter	Value	Parameter	Value
$m_{a}$	34 (kg)	$m_{f}$	4 (kg)
$m_{b}$	3 (kg)	$m_{d}$	3 (kg)
$m_{h}$	51	$\rho_{h}$	(−0.03, 0, −0.09) (m)
$\rho_{g}$	(0.5351, 0, 0.1275) (m)	$\rho_{f}$	(0.0261, 0, 0.2188) (m)
$\rho_{a}$	(0.5801, 0, 0.2625) (m)	$\rho_{d}$	(0.0321, 0, 0.5887) (m)
$\rho_{b}$	(−0.3649, 0, 0.5275) (m)	$\rho_{e}$	(0.4427, 0, −0.0725) (m)
*ε*	18°	$r_{r}$	0.3 (m)
*g*	9.81 (m/s^2^)	$r_{f}$	0.35 (m)
$k_{\gamma }$	951 (Nm/rad)	$c_{\gamma }$	42.2 (Nms/rad)
	(1)	*-*	-

**Table 2:** Mass moment of inertia for the bicycle–rider model.

Part	Moment of Inertia (kg·m^2^)
Vehicle body	$I_{A}=\left[\begin{array}{ccc} 3.8690 & 0 & 1.3\\ & 4.667 & 0\\ & & 1.272 \end{array}\right]$
Front fork	$I_{F}=\left[\begin{array}{ccc} 0.05892 & 0 & -0.00756\\ & 0.06 & 0\\ & & 0.00708 \end{array}\right]$
Rear wheel	$I_{B}=\left[\begin{array}{ccc} 0.0603 & 0 & 0\\ & 0.12 & 0\\ & & 0.0603 \end{array}\right]$
Front wheel	$I_{D}=\left[\begin{array}{ccc} 0.1405 & 0 & 0\\ & 0.28 & 0\\ & & 0.1405 \end{array}\right]$
Rider’s upper body	$I_{A}=\left[\begin{array}{ccc} 4.299 & 0 & 1.444\\ & 5.186 & 0\\ & & 1.413 \end{array}\right]$

**Table 3:** Simulation parameters for a bicycle–rider with an ASAS.

Parameter	Value	Unit
$m_{a}$	34	kg
$\rho_{a}$	(0.5801, 0, 0.2625)	m
$I_{A}$	$I_{A}=\left[\begin{array}{ccc} 3.8690 & 0 & 1.3\\ & 4.667 & 0\\ & & 1.272 \end{array}\right]$	kg·m^2^
$\rho_{g1}$	(0.1251, 0, 0.1266)	m
$\rho_{g2}$	(0.3851, 0, 0.1266)	m
